# Supplementary material for: “Organ-in-a-Column” Coupled On-line with Liquid Chromatography-Mass Spectrometry
Source: Anal Chem. 2022 Dec 9;94(50):17677–84. doi: 10.1021/acs.analchem.2c04530 (PMC9773175; doi:10.1021/acs.analchem.2c04530)
Supplement: Supplementary file 1 — ac2c04530_si_001.pdf [file ac2c04530_si_001.pdf]

Supporting Information for:
"Organ-in-a-column" coupled on-line with liquid chromatography-mass spectrometry

Stian Kogler[1,2], Aleksandra Aizenshtadt[1], Sean Harrison[1,3], Frøydis Sved Skottvoll[1,2], Henriette Engen Berg[2], Shadab Abadpour[1,4], Hanne Scholz[1], Gareth Sullivan[3,5], Bernd Thiede[6], Elsa Lundanes[2], Inger Lise Bogen[7], Stefan Krauss[1,8], Hanne Røberg-Larsen[2], Steven Ray Wilson[1,2]*

[1] Hybrid Technology Hub - Centre of Excellence, Institute of Basic Medical Sciences, Faculty of Medicine, University of Oslo, P.O box 1110 Blindern 0317 Oslo, Norway
[2] Section for Chemical Life Sciences, Department of Chemistry, University of Oslo, P.O box 1033 Blindern 0315 Oslo, Norway
[3] Department of Pediatric Research, Oslo University Hospital, P. O. Box 4950 Nydalen, N-0424 Oslo, Norway
[4] Department of Transplant Medicine and Institute for Surgical Research, Oslo University Hospital, Rikshospitalet, P. O. Box 4950 Nydalen, 0424 Oslo, Norway
[5] Institute of Immunology, Oslo University Hospital, P. O. Box 4950 Nydalen Oslo, Norway
[6] Section for Biochemistry and Molecular Biology, Department of Biosciences, University of Oslo, P. O. Box 1066 Blindern 0316 Oslo, Norway
[7] Section for Drug Abuse Research, Department of Forensic Sciences, Oslo University Hospital, P. O. Box 4950 Nydalen Oslo, Norway
[8] Department of Immunology and Transfusion Medicine, Oslo University Hospital, Rikshospitalet, P. O. Box 4950 Nydalen Oslo, Norway

*Corresponding author: Steven Ray Wilson, stevenw@kjemi.uio.no, +47 97010953. Full address: Department of Chemistry, University of Oslo, P. O. Box 1033, Blindern, NO-0315 Oslo, Norway. https://orcid.org/0000-0002-9755-1188

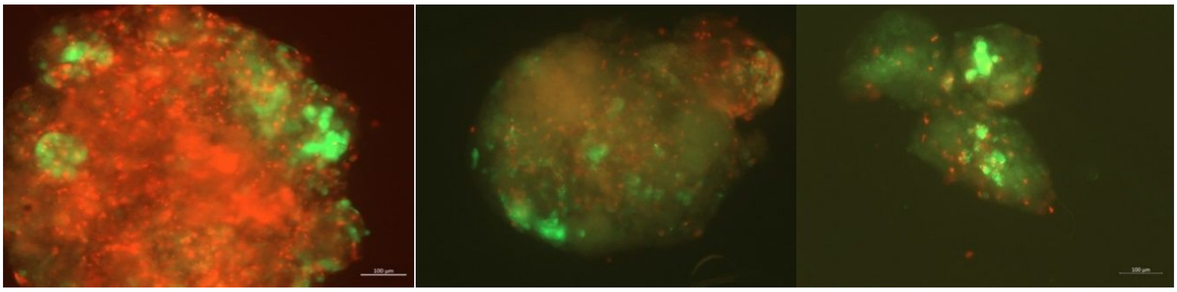

Figure SI1. Live/dead staining of liver organoids flushed from the LC column after 7 days on-line experiments. Live cells were stained green. Most (>80%) organoids showed low amounts of dead staining (red color, middle, right) even after exposure to high concentrations of heroin and left under perfusion of medium without oxygenation for more than 7 days. However, some in-column variation was seen with some organoids showing >90% dead stain (left). Scale bar is 100 µm.

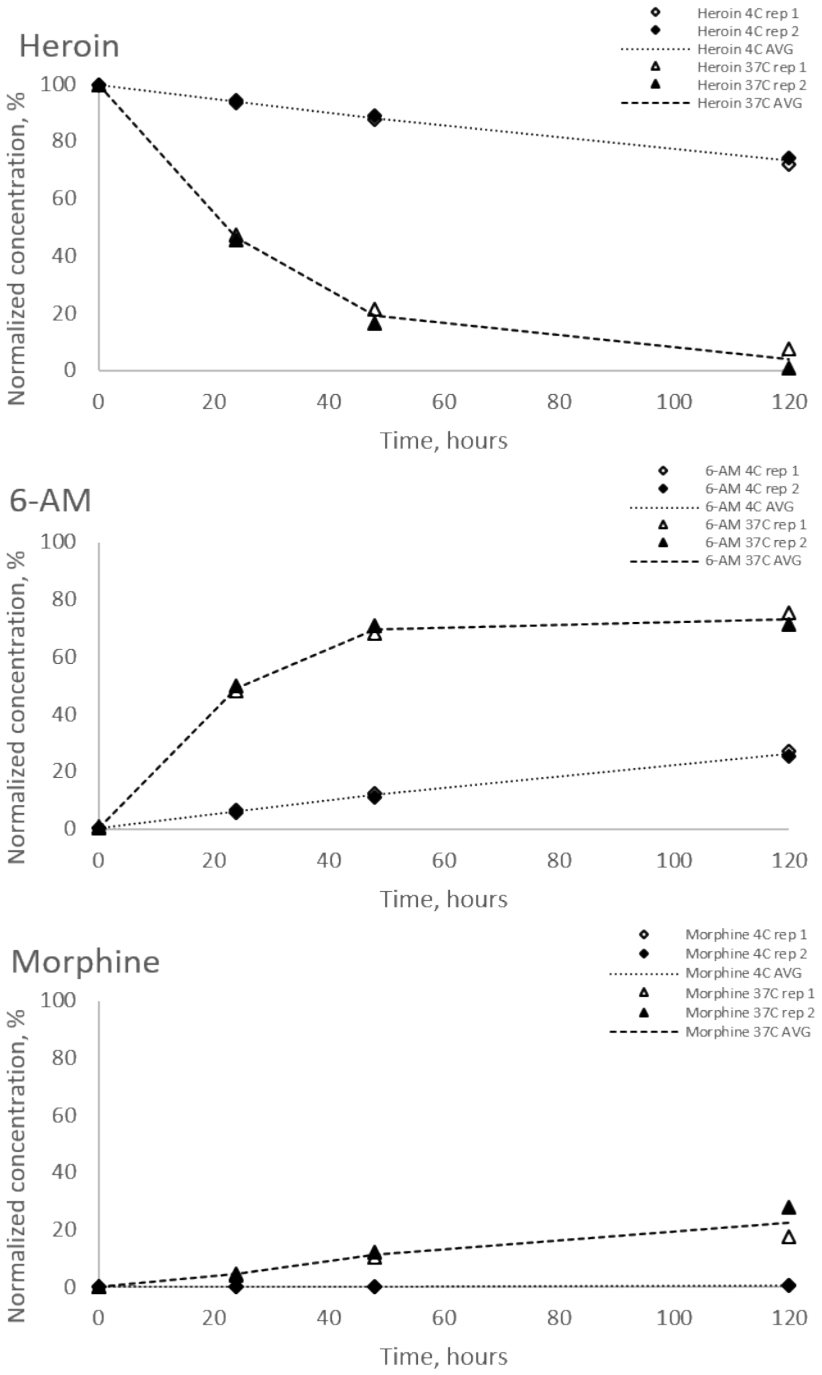

Figure SI2. Heroin stability testing: Spontaneous cell-independent degradation of heroin (10 µM) formation of 6-AM and morphine from heroin at different temperatures in serum free organoid medium (4 ˚C vs 37 ˚C).

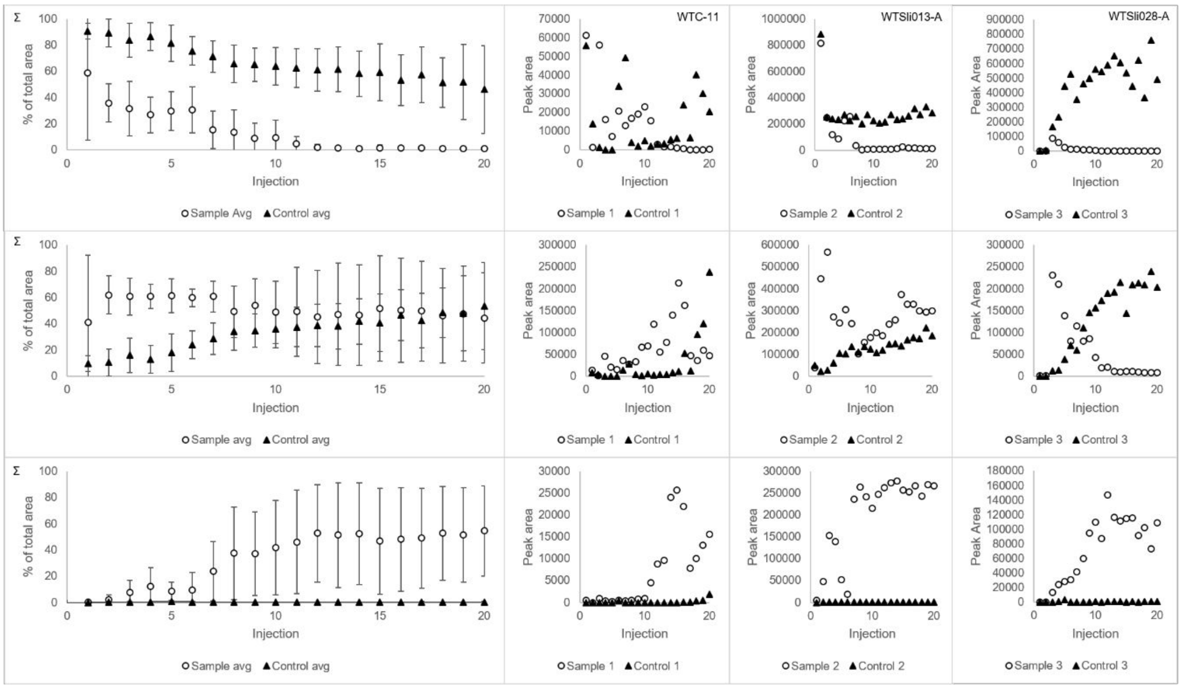

Figure SI3. On-line enzymatic and non-enzymatic conversion of heroin (10 µM) to 6-AM and morphine in columns containing approximately 50 organoids (sample) and columns containing no organoids (control). The top row shows average areas of heroin and metabolites normalized to the average total area of heroin, 6-AM and morphine (area-% of avg. total area of heroin, 6-AM, and morphine). The average total areas of heroin, 6-AM and morphine (top row) are based on three experiments performed on different days (10 h experiments) and columns, with three different cell lines displayed with the name of the cell line.

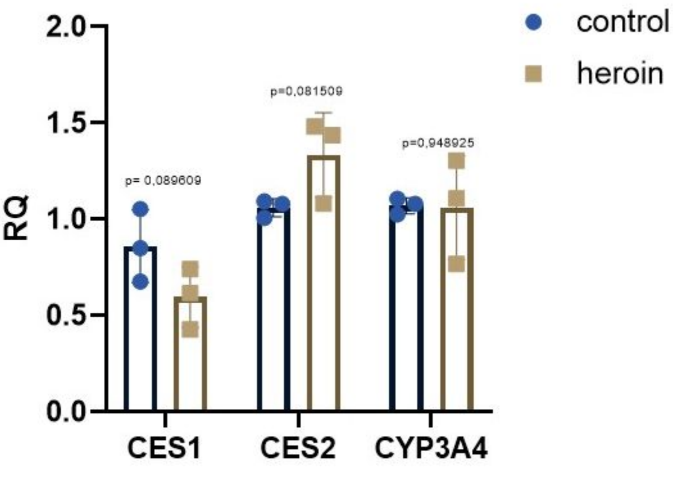

Figure SI4. Relative expression of CES1, CES2 and CYP3A4 in heroin-treated and control iHLC organoids. Significance was calculated using unpaired t-test.

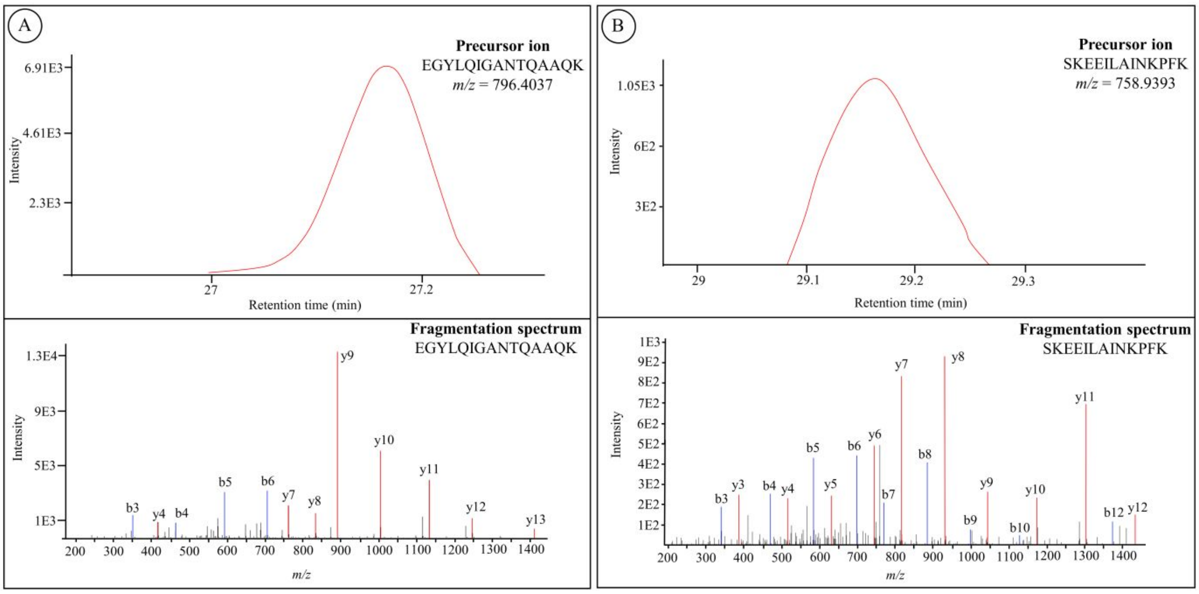

Figure SI5. Detection of human liver carboxylesterase 1 (CES1, **A**) and human liver carboxylesterase 2 (CES2, **B**) in liver organoids. The example shown in this figure is iHLC generated from WTC-11 cell line, treated with heroin (10 µM). The figure shows the extracted ion chromatograms (top) of the unique tryptic peptides EGYLQIGANTQAAQK (for CES1) and SKEEILAINKPFK (for CES2) and the fragmentation spectrums (bottom) with detected y and b ions marked in red and blue, respectively. The peptides were separated using a 25 cm x 75 µm IonOpticks column (1.6 µm silica particles). The mobile phases contained 0.1% formic acid in water (A) and 0.1% formic acid in acetonitrile (B). A linear gradient from 0-35% mobile phase B over 54 min at a flow rate of 300 nL/min at a column temperature of 50 °C was employed. MS acquisition was performed using a timsTOF in data-dependent acquisition parallel accumulation-serial fragmentation (DDA-PASEF) mode. Detection was performed with at least 1 unique peptide and a false discovery rate at ≤1%.
